# Proximal Articular Set Angle Correction with the Reverdin–Isham Osteotomy in Mild and Moderate Hallux Valgus: A Systematic Review and Meta-Analysis

**DOI:** 10.3390/medicina61030406

**Published:** 2025-02-26

**Authors:** Diego Mosquera-Canosa, Eduardo Nieto-García, Óscar Álvarez-Calderón-Iglesias, Rubén García-Fernández, Miguel López-Vigil, Hector Pereiro-Buceta, Roi Painceira-Villar, Natalia Calvo-Ayuso, Bibiana Trevissón-Redondo

**Affiliations:** 1 PhD School, Universidad de León, 24401 León, Spain; dmosqc00@estudiantes.unileon.es; 2 Department of Podiatry, School of Medicine and Health Sciences, Universidad Católica de Valencia “San Vicente Mártir”, 46001 Valencia, Spain; eduardo.nieto@ucv.es; 3 Faculty of Nursing and Podiatry, Fundación HM de Investigación, 28015 Madrid, Spain; oscaraci.udc@gmail.com; 4 SALBIS Research Group, Faculty of Health Sciences, Department of Nursing and Physiotherapy, Campus de Ponferrada, Universidad de León, 24401 León, Spain; ncala@unileon.es (N.C.-A.); btrer@unileon.es (B.T.-R.); 5 Nursing Research, Innovation and Development Centre of Lisbon (CIDNUR), Nursing School of Lisbon, 1600-190 Lisbon, Portugal; 6 Department of Nursing and Physiotherapy, Campus de Ponferrada, Universidad de León, 24401 León, Spain; mlopv@unileon.es (M.L.-V.); rpaiv@unileon.es (R.P.-V.)

**Keywords:** Hallux valgus, Hallux abductus valgus, Reverdin–Isham osteotomy, oblique osteotomy, minimally invasive surgery, PASA, proximal articular angle, DMAA, distal metatarsal articular angle, AOFAS scale

## Abstract

*Background and Objectives:* The Reverdin–Isham osteotomy via minimal incision is a prominent option for the surgical treatment of hallux valgus, a foot deformity characterized by medial deviation of the first metatarsal and lateral deviation of the first toe. This technique is particularly indicated for cases with an elevated proximal articular angle, enabling effective correction and improved foot functionality. However, its efficacy has not been fully established in the scientific literature. Objective: The main objective of this analysis was to evaluate the correction of radiological angles (PASA, IMA, and HVA), the improvement in functionality according to the AOFAS scale, pain reduction, and patient satisfaction. *Materials and Methods:* A systematic review was conducted following the PRISMA guidelines in scientific databases such as PubMed, Scopus, and Web of Science, assessing publication biases and heterogeneity. Ten studies were included, covering 579 procedures in 500 patients, with an average follow-up of 33.8 months. *Results:* The results did not show significant improvements in the PASA (*p* = 0.14; CI [−1.52, 0.32]), not in the AIM (*p* = 0.05; CI [−2.63, 0.02]), although the meta-regression was statistically significant (*p* = 0.0022) with a ratio of 61.2%. It did show significant improvements in the AHV (*p* = 0.0009; CI [−3.14, −1.33]). An increase of 37.4 points in the AOFAS scale was revealed, and a 5.4-point reduction in pain. Patient satisfaction was high, with 89.3% of patients satisfied and 94.7% willing to undergo the procedure again. However, 20 major complications were reported, primarily recurrences. *Conclusions:* The Reverdin–Isham osteotomy is a safe and effective technique for treating mild-to-moderate hallux valgus. It offers significant improvements in foot functionality and pain reduction, with high patient satisfaction rates. Although recurrences remain the main complication, their low frequency reinforces the validity of the technique as a surgical option for specific deformities.

## 1. Introduction

Hallux valgus (HV) deformity is one of the most common pathologies of the forefoot, characterized by a progressive lateral deviation of the proximal phalanx relative to the first metatarsal. This results in articular incongruence between the base of the first phalanx and the first metatarsal caused by the pronation of the first toe. Individuals affected often experience chronic pain, discomfort, and deterioration in performing basic daily activities [1]. Surgical intervention is indicated when conservative strategies, such as footwear modifications and customized orthoses, fail to alleviate or reduce symptoms [2].

More than 150 surgical techniques have been documented to treat HV, yet no consensus has been reached on a single optimal or “gold standard” procedure due to the variability among patients. The Reverdin–Isham osteotomy, proposed by Dr. Stephen Isham in 1985, is a modification of the Reverdin technique. It employs a medial wedge oblique osteotomy while preserving the lateral cortex of the first metatarsal head and is associated with minimally invasive surgical (MIS) approaches [3].

Research has shown a positive correlation between HV deformity and an increased proximal articular set angle (PASA), also known as the distal metatarsal articular angle (DMAA) [4]. This indicates that the lateral inclination of the metatarsal head contributes to the deformity [5]. The Reverdin–Isham osteotomy has been identified as an effective technique for correcting this angle.

The diagnosis of HV is primarily based on clinical presentation, physical examination, family history, and radiological studies. Various radiological angles must be considered, including weight-bearing anteroposterior, lateral, and oblique projections.

Several classifications for HV have been proposed, with one of the most commonly used being based on parameters such as the hallux valgus angle (HVA), intermetatarsal angle (IMA), and the degree of sesamoid subluxation. These classifications and the severity of HV in a specific patient guide the selection of protocols and surgical interventions based on the magnitude and characteristics of the deformity [6].

Conservative strategies include appropriate footwear, customized plantar orthoses, non-steroidal anti-inflammatory drugs, or physiotherapy [7]. Surgical intervention is considered when conservative methods fail to provide satisfactory results. While conservative management may offer short-term relief, it rarely suffices in the long term [8]. Studies suggest that surgical correction is more effective in reducing pain, improving aesthetics, and enabling a return to normal daily activities compared to conservative approaches [8].

Minimally invasive techniques for HV treatment offer substantial advantages over traditional open surgery. Notable benefits, supported by studies, include reduced soft tissue damage, shorter recovery time, minimized surgical time and postoperative pain, and significant radiological correction of the deformity. Patient satisfaction is comparable to that of open surgery [9,10,11,12].

One specific MIS technique is the Reverdin–Isham osteotomy. This intracapsular technique is used to treat mild-to-moderate hallux valgus deformity [13,14,15]. It aims to correct the hallux alignment and improve foot function [15]. The procedure involves a minimally invasive approach using small skin portals of 2–4 mm. Initially, a dorsolateral bunionectomy is performed to remove the abnormal prominence of the first metatarsal head, followed by lateral release of the adductor hallucis muscle. Next, a wedge osteotomy is performed while preserving the lateral cortex. The medial base and lateral apex wedge are then closed under fluoroscopic control, correcting the deformity [13].

The current scientific literature on hallux valgus presents significant limitations that hinder a comprehensive understanding of the condition. One of the main shortcomings is the lack of standardized classification criteria, which complicates the comparison of results across studies and restricts the applicability of findings. Moreover, most available studies face methodological limitations, such as small sample sizes, retrospective designs, or the absence of control groups, thereby affecting the validity of the results.

Additionally, there is an insufficiency of studies addressing long-term outcomes, leaving unresolved questions regarding the durability of corrections, future complications, and recurrence rates. This is compounded by the lack of detailed analyses on the overall impact on patients’ quality of life, an essential aspect that remains underexplored. Furthermore, the analysis of complications and predictive factors of treatment success for hallux valgus is limited, making it challenging to tailor therapeutic approaches to individual patients.

In general, current research faces challenges related to the heterogeneity of studied populations, the predominant focus on short-term outcomes, and the underrepresentation of non-surgical interventions. These deficiencies highlight the need for more rigorous, multicenter studies with a comprehensive approach to advance the optimal management of hallux valgus.

## 2. Materials and Methods

Following the PICO Methodology (Population, Intervention, Comparison, Outcome):

(P) Population: Adult patients with hallux valgus.

(I) Intervention: Performance of the Reverdin–Isham osteotomy.

(C) Comparison: Patients undergoing surgery for mild-to-moderate hallux valgus with the Reverdin–Isham osteotomy.

(O) Outcome: Efficacy of the osteotomy in terms of angular correction, pain reduction, satisfaction, complications, and first ray functionality.

The following research question was formulated:

What is the efficacy of the Reverdin–Isham osteotomy in correcting the PASA in mild-to-moderate hallux valgus?

### 2.1. Identification and Selection of Research Studies

Search Strategy:

A bibliographic search was conducted in December 2023 and January 2024 using the PubMed, SCOPUS, and Web of Science (WOS) databases. The search strategy and the results obtained after applying filters in each database are summarized in Table 1. This systematic review adhered to the 2020 PRISMA guidelines (Preferred Reporting Items for Systematic Reviews and Meta-Analyses) [16]. The review was submitted to the PROSPERO registry (registration number CRD42024549529) to prevent content duplication, minimize bias, and enable review comparisons. Additionally, a protocol was registered on the Open Science Foundation (OSF) platform (https://osf.io/2yg3z, accessed on 11 December 2024).

### 2.2. Selection and Exclusion Criteria

Inclusion criteria for studies in this systematic review:

- Adult patients diagnosed with hallux valgus.
- Use of the Reverdin–Isham osteotomy as the primary surgical technique, alone or in combination with other techniques.
- Publication dates within the last 10 years (2013–2023).
- Studies published in English or Spanish.

Exclusion criteria for studies:

- Studies conducted on cadaver specimens.
- Studies that did not report any radiological angle data.
- Animal studies.
- Systematic reviews or studies with sample sizes of fewer than ten cases to avoid bias and ensure statistically robust and representative results.
- Letters to the editor or expert opinions.

**Figure 1:**
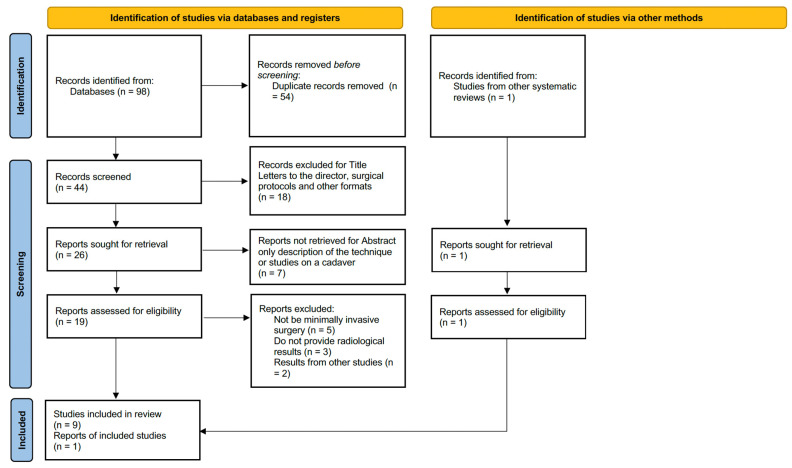
PRISMA flowchart for study selection.

The selection of articles for this systematic review followed a rigorous process. Using the Zotero bibliographic reference manager (v 6.0.30), duplicate records were first removed. Subsequently, a sequential filtering process was applied, including title screening, abstract review, and full-text reading to determine eligibility based on predefined inclusion criteria. In cases where abstracts did not provide sufficient information, full-text articles were reviewed. The flowchart (Figure 1) illustrates the selection process.

The initial search was conducted in PubMed, SCOPUS, and WOS using specific keywords and Boolean operators, resulting in the identification of 98 studies. After removing duplicates, 44 studies were evaluated, of which 26 were selected for abstract review. Seven studies were excluded because they only described the technique or were based on cadaveric samples. After full-text review, an additional 10 studies were excluded for not meeting the minimally invasive criteria, referencing results from other studies rather than their own, or failing to provide radiological data. This yielded nine final studies for detailed review.

One additional study, not initially identified through the keyword search, was incorporated as referenced in other related systematic reviews. As a result, 10 articles were included for detailed analysis.

The following variables from the studies included in the systematic review were selected and summarized in Table 2 and Table 3:

- Author and year.
- Study design.
- Study duration.
- Sample characteristics (number of patients, operated feet, mean age, sex).
- Follow-up period (in months).
- Associated surgical techniques.
- Outcomes related to angular correction, pain, satisfaction, and major complications.

### 2.3. Evaluation of Study Quality

**Figure 2:**
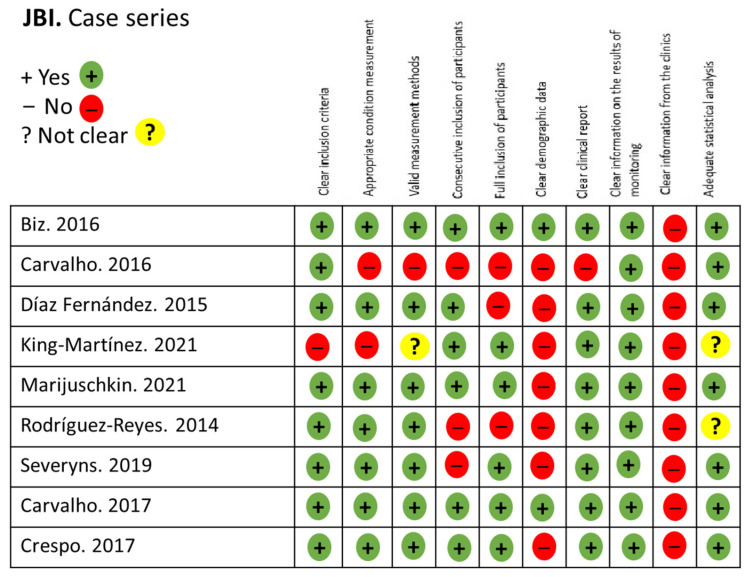
JBI for case series studies [6,19,20,22,23,24,25,26,27].

**Figure 3:**
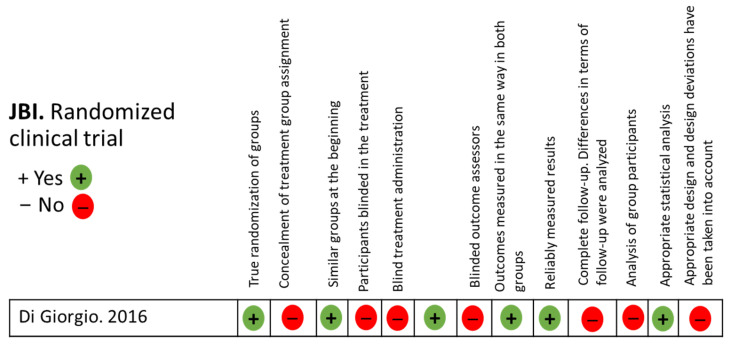
JBI for randomized clinical trial studies [21].

The quality and risk of bias of the studies were assessed using the Joanna Briggs Institute (JBI) critical appraisal tools [17], tailored to the type of study analyzed. A quantitative analysis was performed using different checklists that evaluated predefined criteria. Each item was categorized as “No” (−; high risk of bias), “Unclear” (?; moderate risk of bias), “Yes” (+; low risk of bias), or “Not Applicable” (NA) as appropriate (Figure 2 and Figure 3).

**Figure 4:**
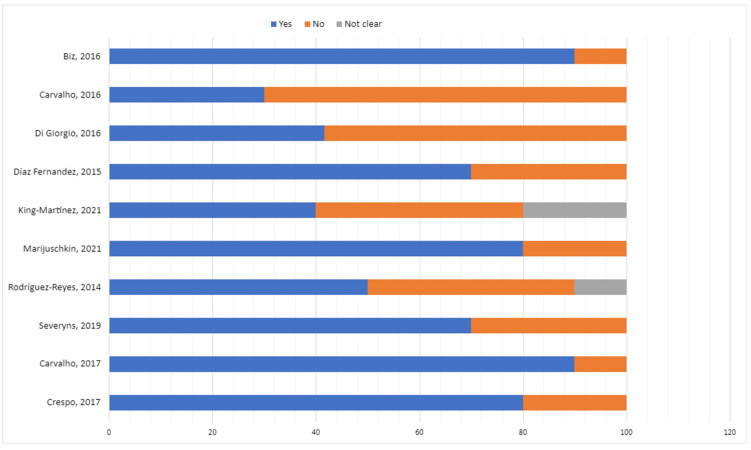
Risk of bias for studies included in the review [6,19,20,21,22,23,24,25,26,27].

Studies were classified as follows:

- Low risk of bias: >70% “Yes” responses.
- Moderate risk of bias: 50–70% “Yes” responses.
- High risk of bias: <50% “Yes” responses. (Figure 4).

To determine the recommendation strength and evidence level of the selected articles, the Oxford Centre for Evidence-Based Medicine (CEBM) scale was applied [18] (Table 4).

**Table 2:** Summary of variables from analyzed studies.

Study	Design	Temporality	Sample	Follow-Up	Associated Technique
Biz et al. 2016 [19]	Prospective Case Series	May 2010– May 2012	80 patients. 80 feet (43 Right, 37 Left). Mean age 51 years. 75 Women, 5 Men.	48 months	Adductor tenotomy, capsulotomy, and Akin
Carvalho et al. 2016 [20]	Prospective Case Series	December 2005– March 2009	61 patients. 93 feet. Group 1 (29) unilateral. Group 2 (32) bilateral. Mean Age 61.5 years (G1) 58.7 years (G2). 29 Women (G1) 2 Men 30 Women (G2)	24 months G1 28 months G2	Adductor tenotomy and Akin
Di Giorgio et al. 2016 [21]	RCT	February 2011– January 2013	19 patients. Mean age 40.8 years	22 months	Adductor tenotomy, capsulotomy, and Akin
Díaz Fernández et al. 2015 [22]	Prospective Case Series	May 2009– March 2013	41 patients. 44 feet (3 deferred bilaterals). Mean age 61 years. 36 Women, 5 Men.	24 months	Adductor tenotomy, capsulotomy, Akin, and osteotomy in base
King-Martínez et al. 2021 [23]	Case Series	January 2008– December 2008	36 patients (27 bilateral) 63 feet. Mean age 49 years. 34 Women, 2 Men	24 months	Adductor tenotomy, capsulotomy, and Akin
Marijuschkin et al. 2021 [6]	Prospective Case Series	One year	72 patients, 112 feet (35 Reverdin)	36 months	Adductor tenotomy, capsulotomy, and Akin
Rodríguez-Reyes et al. 2014 [24]	Case Series	-	11 patients. 20 feet (9 bilateral). Mean Age 46.9 years 10 Women, 1 Man	6 months radiography 12 months AOFAS (American Orthopaedic Foot Scale and Ankle Society)	-
Severyns et al. 2019 [25]	Case Series	May 2003–November 2011	48 patients, 57 feet (9 bilateral) Mean Age 51.5 years. 43 Women, 5 Men.	60.1 months	Adductor tenotomy, capsulotomy, and Akin
Carvalho et al. 2017 [26]	Prospective Case Series	2006–2009	25 patients. 36 feet (17 Left 19 Right) Mean Age 57.5 years 21 Women, 4 Men	64.8 months	Adductor tenotomy, capsulotomy, and Akin
Crespo et al. 2017 [27]	Prospective Case Series	April 2006–December 2013	108 patients. 132 feet. (24 bilateral) Mean age 56.1 years. 7 Men, 125 Women.	24 months	Adductor tenotomy, capsulotomy, Akin, and 36 base osteotomies

**Table 3:** Summary of results from analyzed studies.

Study	Angular Correction	Pain	Satisfaction	Major Complications
Biz et al. 2016 [19]	PASA presurgical 10.12° postsurgical 5.4° AIM presurgical 12.9° postsurgical 9° HAV presurgical 26.4° postsurgical 13.9°	AOFAS presurgical 54.1. AOFAS postsurgical 87.1	-	5 recurrences. 1 severe stiffness.
Carvalho et al. 2016 [20]	Group 1 PASA presurgical 13.5° postsurgical 6° AIM presurgical 14.4° postsurgical 13.6° AHV presurgical 32.5° postsurgical 19.4° Group 2 PASA presurgical 16.1° postsurgical 8.1° AIM presurgical 13.4° postsurgical 12.4° HAV presurgical 34.1° postsurgical 19.2°	AOFAS postsurgical Group 1 82.9. AOFAS postsurgical Group 2 86.8	Satisfied or Very Satisfied Group 1: 90.6% Group 2: 89.7%	-
Di Giorgio et al. 2016 [21]	AIM presurgical 14.1° postsurgical 8.9° HAV presurgical 30.2° postsurgical 13.1°	AOFAS presurgical 40.5 AOFAS postsurgical 90.3	100% satisfied or satisfied with reservations 94.7% Would Undergo Surgery Again	-
Díaz Fernández et al. 2015 [22]	PASA presurgical 16.09° postsurgical 9.52° AIM presurgical 16.88° post surgica l8.18° HAV presurgical 40.02° postsurgical 10.51°	AOFAS presurgical 48.14. AOFAS postsurgical 91.28.	-	3 recurrences. 1 deep infection
King-Martínez et al. 2021 [23]	PASA presurgical 13° postsurgical 8.27° AIM presurgical 13.81° postsurgical 11.83° HAV presurgical 29.44° postsurgical 13.59°	AOFAS presurgical 49.24 AOFAS postsurgical 94.32. VAS (Visual Analogic Scale) presurgical 8.02 VAS postsurgical 1.49	Satisfied or Very Satisfied 87.3%	-
Marijuschkin et al. 2021 [6]	AIM presurgical 13.4° postsurgical 12.6° HAV presurgical 26.5° postsurgical 13.7°	AOFAS presurgical 52.4 AOFAS postsurgical 85.3		2 case of joint stiffness.
Rodríguez-Reyes et al. 2014 [24]	PASA presurgical 9.1° postsurgical 5.3° AIM presurgical 9.7° postsurgical 9.5° HAV presurgical 24.8° postsurgical 15.5°	AOFAS presurgical 60.5 AOFAS postsurgical 95.7		-
Severyns et al. 2019 [25]	PASA presurgical 14.1° postsurgical 7.7° AIM presurgical 13.5° postsurgical 12° HAV presurgical 29.3° postsurgical 15.5°	AOFAS presurgical 55.9 AOFAS postsurgical 89.2	Satisfied or Very Satisfied 89.5%	1 TVP case, 2 recurrences
Carvalho et al. 2017 [26]	PASA mean correction 9.3° AIM mean correction 2.7° HAV mean correction 14.6°	AOFAS postsurgical mean 88.6	Satisfied or Very Satisfied 91.7%	5 recurrences
Crespo et al. 2017 [27]	PASA presurgical 18.5° postsurgical 23.6° AIM presurgical 13.1° postsurgical 10.7° HAV presurgical 34.3° postsurgical 22.5°	AOFAS presurgical 50.6 AOFAS postsurgical 85.9. VAS presurgical 6.3. VAS postsurgical 1.9.	Satisfied or Very Satisfied 76.5%	-

**Table 4:** Levels of evidence and grades of recommendation (Oxford).

Study	Grade of Recommendation	Level of Evidence	Source
Biz. 2016 [19] Carvallho. 2016 [20]	Favorable but Inconclusive Recommendation	4	Case Series
Di Giorgio. 2016 [21]	Favorable Recommendation	2b	Low-Quality RCT
Díaz Fernández. 2015 [22] King-Martínez. 2021 [23] Marijuschkin. 2021 [6] Rodríguez-Reyes. 2014 [24] Severyns. 2019 [25] Carvalho. 2017 [26] Crespo. 2017 [27]	Favorable but Inconclusive Recommendation	4	Case Series

After an exhaustive analysis of the studies, the key findings are summarized as follows:

The study of Biz et al. [19] addressed the Reverdin–Isham osteotomy combined with adductor tenotomy and lateral capsulotomy in 80 patients, totaling 80 operated feet. Significant improvements were observed in the three angles studied, with a mean correction of 4.72° for PASA, 3.9° for IMA, and 12.5° for HVA. Additionally, an average improvement of 33 points was recorded on the AOFAS scale (American Orthopaedic Foot Scale and Ankle Society). However, 5 cases of recurrence and 1 case of severe joint stiffness were reported as major complications.

Carvalho et al. [20] involved 61 patients divided into two groups: one operated on a single foot (Group 1) and the other on both feet (Group 2), totaling 90 operated feet. The Reverdin–Isham osteotomy was combined with adductor tenotomy and Akin osteotomy. Both groups showed significant angular improvements, with Group 1 experiencing a mean correction of 7.5° for PASA, 0.7° for IMA, and 13.2° for HVA, and Group 2 showing a mean correction of 8° for PASA, 1° for IMA, and 14.8° for HVA. However, no data on the AOFAS scale were provided for either group.

In the study of Di Giorgio et al. [21], a comparative clinical trial was conducted between two groups of patients undergoing HV surgery: one group underwent the Reverdin–Isham osteotomy, and the other the Endolog technique. The Reverdin–Isham group included 19 unilaterally operated patients, combining osteotomy with adductor tenotomy, lateral capsulotomy, and Akin osteotomy. Although PASA was not analyzed, significant angular improvements were observed in IMA (5.2°) and HVA (17.1°). A notable improvement of 49.8 points was recorded on the AOFAS scale, the highest among the reviewed studies.

The study of Díaz Fernández et al. [22] involved 41 patients, including 3 who underwent bilateral surgery at different times, totaling 44 Reverdin–Isham osteotomies. The procedure was combined with adductor tenotomy, lateral capsulotomy, Akin technique, and basal osteotomy for more complex HV cases. Significant improvements were observed in all angles evaluated, with a mean correction of 6.56° for PASA, 8.77° for IMA, and 29.22° for HVA—the latter two being the best results obtained in the review. An average improvement of 37.12 points was recorded on the AOFAS scale. Major complications included 3 recurrences and 1 deep infection.

The study of King-Martínez et al. [23] included 36 patients, 27 of whom underwent bilateral surgery, totaling 63 operated feet using the Reverdin–Isham osteotomy combined with adductor tenotomy, capsulotomy, and Akin technique. Mean improvements were 4.73° for PASA, 1.98° for IMA, and 15.85° for HVA. Additionally, a mean improvement of 45.08 points was recorded on the AOFAS scale.

In the case of the study of Marijuschkin et al. [6], they evaluated four intervention groups based on HV severity. The group treated with the Reverdin–Isham osteotomy included 35 feet, combined with adductor tenotomy, lateral capsulotomy, and Akin osteotomy. While no PASA data were available, mean corrections of 0.8° for IMA and 12.8° for HVA were observed. The AOFAS scale showed an average improvement of 32.9 points. Notable complications included two cases of joint stiffness.

The study of Rodríguez-Reyes et al. [24] evaluated plantar pressures and angular radiological analyses before performing the Reverdin–Isham osteotomy. The sample included 11 patients, 9 of whom underwent bilateral surgery. Mean angular corrections were 3.8° for PASA, 0.2° for IMA, and 13.9° for HVA. An average improvement of 35.2 points was recorded on the AOFAS scale.

Severyns et al. [25] included 48 patients, 9 of whom underwent bilateral surgery, totaling 57 operated feet using the Reverdin–Isham osteotomy combined with adductor tenotomy, lateral capsulotomy, and Akin osteotomy. Mean angular improvements were 6.4° for PASA, 1.5° for IMA, and 13.9° for HVA. An average improvement of 33.3 points was recorded on the AOFAS scale. Major complications included one case of deep vein thrombosis and two recurrences.

The study of Carvalho et al. [26] (2017) included 25 patients, with 36 operated feet undergoing the Reverdin–Isham osteotomy combined with adductor tenotomy, lateral capsulotomy, and Akin osteotomy. Mean corrections were 9.3° for PASA, 2.7° for IMA, and 14.6° for HVA, achieving the greatest PASA correction among the reviewed studies. Although pre- and postoperative data were limited, the final mean AOFAS score was 88.6 points, but no preoperative score was provided. Five recurrences were reported as major complications.

Crespo et al. [27] included 108 patients and 132 operated feet using the Reverdin–Isham osteotomy combined with adductor tenotomy, lateral capsulotomy, Akin technique, and basal osteotomy in 36 cases. This was the only study reviewed that showed a worsening of the PASA, with a mean deterioration of 5.1°. Improvements of 2.4° for IMA and 11.8° for HVA were observed. The AOFAS scale recorded a mean improvement of 35.3 points.

## 3. Results

The main characteristics of the 10 studies that met the eligibility criteria are summarized concisely in Table 2 and Table 3. The countries of origin of the included studies were Italy [19,21], Portugal [20,26], Spain [22,27], Mexico [23,24], Brazil [6], and France [25]. Most studies (nine out of ten) were case series, with only one low-quality clinical trial [27]. The publication dates of the studies ranged from 2013 to 2024.

The quality assessment revealed a moderate level of quality with a similarly moderate risk of bias, as shown in Figure 2, Figure 3 and Figure 4. Regarding the level of scientific evidence and recommendation grades, a favorable but inconclusive recommendation was established, as illustrated in Table 4.

This systematic review analyzed a total of 500 patients who underwent hallux valgus surgery using the Reverdin–Isham technique. Of these, 403 were women (80.6%), 31 were men (6.2%), and the sex of the remaining 66 was unspecified. The mean age of the patients was 52.65 years (range: 40.8–60.1), with an average follow-up of 33.8 months.

A total of 579 feet underwent the Reverdin–Isham technique, with various combined approaches. Specifically: the technique was combined with adductor tenotomy, lateral capsulotomy, and Akin osteotomy in 386 feet (66.7%); combined with adductor tenotomy and Akin in 93 feet (16.1%); combined with adductor tenotomy, lateral capsulotomy, Akin, and basal osteotomy in 80 feet (13.8%); and performed without any associated techniques in 20 feet (3.4%).

Out of the operated feet, 525 (90.7%) were radiologically evaluated for PASA pre- and post-surgery, while all 579 feet (100%) were evaluated for IMA and HVA. The AOFAS scale was used to assess foot function pre- and post-surgery in 500 patients (100%). Additionally, the VAS (Visual Analogic Scale) scale was used to evaluate pain in 144 patients (28.8%).

It was observed that the PASA had a preoperative mean of 13.8 degrees across the analyzed sample, while in the postoperative period, this mean decreased to 9.2 degrees, indicating an average improvement of 4.6 degrees. Regarding the IMA, a preoperative mean of 13.52 degrees was recorded, which was reduced to a postoperative mean of 10.87 degrees, reflecting an average improvement of 2.64 degrees. On the other hand, the HVA showed a preoperative mean of 30.75 degrees, while in the postoperative period, this mean dropped to 15.75 degrees, demonstrating an average improvement of 15 degrees.

According to the associated techniques, it was observed that in cases where adductor tenotomy, lateral capsulotomy, and Akin osteotomy were performed, an average improvement of 5.25 degrees in PASA, 2.67 degrees in IMA, and 14.41 degrees in HVA was achieved. In situations where only adductor tenotomy and Akin osteotomy were performed, an average improvement of 7.75 degrees in PASA, 0.9 degrees in IMA, and 14 degrees in HVA was obtained. In cases where adductor tenotomy, lateral capsulotomy, Akin osteotomy, and basal osteotomy were performed, a mean correction of 9.9 degrees in PASA, 5.55 degrees in IMA, and 20.6 degrees in HVA was recorded. On the other hand, for patients where no combined techniques were mentioned, the average correction in PASA was 3.8 degrees, 0.2 degrees in IMA, and 9.3 degrees in HVA.

The results concerning pain and perceived functionality were evaluated using the American Orthopaedic Foot and Ankle Society (AOFAS) scale and the Visual Analog Scale (VAS). On the AOFAS scale, a preoperative mean score of 51.4 points was recorded, while the postoperative mean score was 88.8 points, reflecting an average improvement of 37.4 points (40.5–95.7 out of 100).

Regarding the VAS, patients showed a preoperative mean score of 7.16 points, which decreased to 1.7 points in the postoperative period, representing an average improvement of 5.4 points (8.02–1.4 out of 10).

In terms of major complications, recurrences were the most common issue, with 15 cases reported (75%), followed by joint stiffness in 15% (3 cases), one infection (5%), and one case of deep vein thrombosis (5%).

Regarding perceived personal satisfaction, 296 patients (51.1%) were evaluated, showing that 89.3% of the patients were satisfied or very satisfied with the intervention, and 94.7% would be willing to undergo the same procedure again.

### 3.1. Statistical Results

#### 3.1.1. PASA

**Figure 5:**
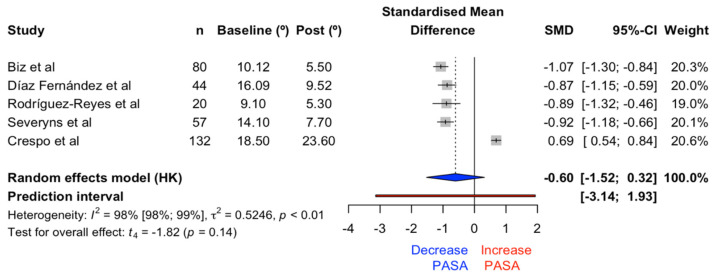
Forest plot for the PASA [19,22,24,25,27].

**Figure 6:**
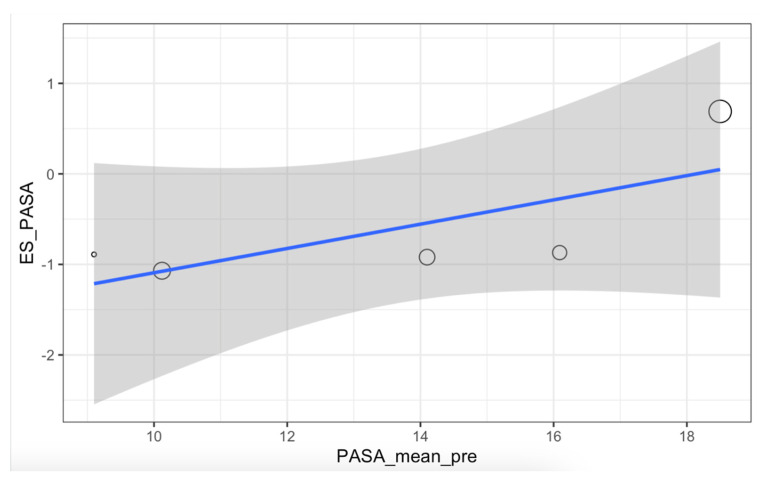
Meta-regression for the PASA.

The meta-analysis on the effects of PASA correction with the Reverdin–Isham osteotomy showed no significant differences (t = −1.82, *p* = 0.14) in favor of reducing the angle, with an effect size of −0.60 (CI: −1.52, 0.32). The forest plot of the PASA and its prediction interval is shown in Figure 5. Heterogeneity index: 98.4% (CI: 97.6%, 98.9%). Meta-regression for PASA was not statistically significant (*p* = 0.06) and the relationship between preoperative angle and the achieved effect was 39% (Figure 6).

**Figure 7:**
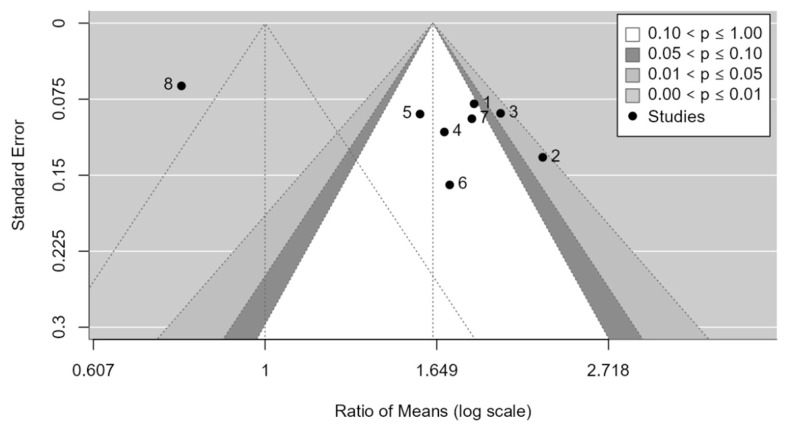
Funnel plot for the PASA.

As shown in the funnel plot (Figure 7), studies are distributed across all zones of significance, suggesting that publication bias is apparently not present. Egger’s test confirms the absence of asymmetry in the funnel plot (*p*-value = 0.0957). No evidence of publication bias was detected.

#### 3.1.2. Intermetatarsal Angle (IMA)

**Figure 8:**
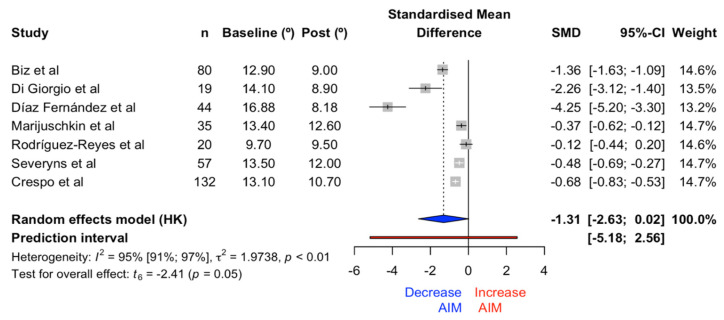
Forest plot for the IMA [6,19,21,22,24,25,27].

The meta-analysis on the effects of the IMA with the Reverdin–Isham osteotomy showed no significant differences (t = −2.41, *p* = 0.05) in favor of angle reduction, with an effect size of −1.31 (CI −2.63, 0.02). The forest plot for IMA is illustrated in Figure 8. The heterogeneity index was established at 94.7% (CI 91.3%, 96.8%).

**Figure 9:**
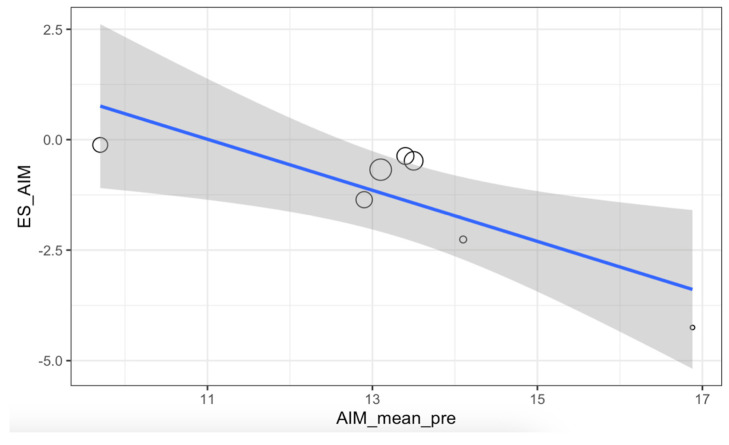
Meta-regression for the IMA.

The meta-regression for the IMA showed statistically significant results (*p* = 0.0022), with the relationship between the preoperative angle and the achieved effect established at 61.2% (Figure 9).

**Figure 10:**
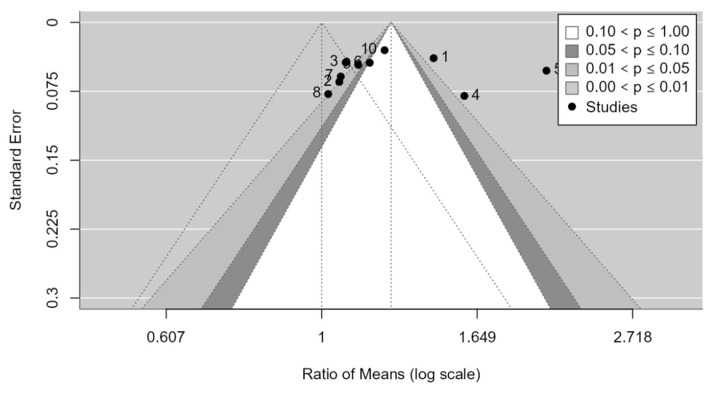
Funnel plot for the IMA.

As shown in the funnel plot (Figure 10), studies are distributed across all zones of significance, suggesting that publication bias is apparently not present. Egger’s test confirms the absence of asymmetry in the funnel plot (*p*-value = 0.4127). No evidence of publication bias was detected.

#### 3.1.3. Hallux Valgus Angle (HVA)

**Figure 11:**
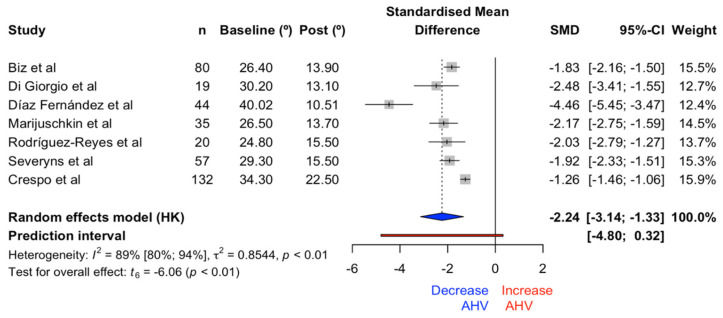
Forest plot for the HVA [6,19,21,22,24,25,27].

The statistical results on the effects of the HVA with the Reverdin–Isham osteotomy showed significant differences (t = −6.06, *p* = 0.0009) in favor of angle reduction, with an effect size of −2.24 (CI −3.14, −1.33). The forest plot for HVA is illustrated in Figure 11. The heterogeneity index was established at 89.3% (CI 80.4%, 94.1%).

**Figure 12:**
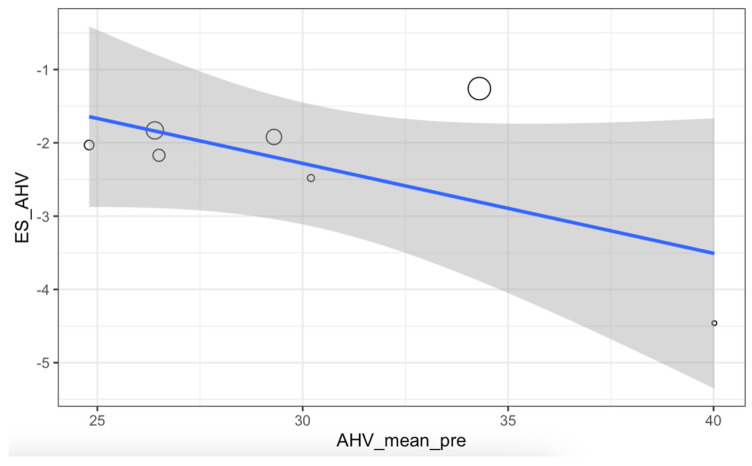
Meta-regression for the HVA.

The meta-regression for HVA did not show statistically significant results (*p* = 0.12), with the relationship between the preoperative angle and the achieved effect established at 12.22% (Figure 12).

**Figure 13:**
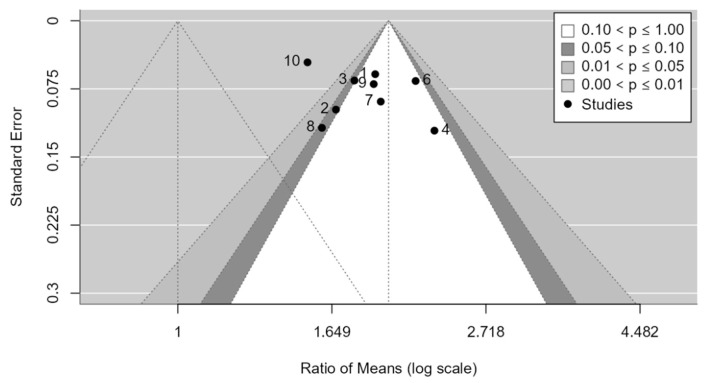
Funnel plot for the HVA.

As shown in the funnel plot (Figure 13), studies are distributed across all zones of significance, suggesting that publication bias is apparently not present. Egger’s test confirms the absence of asymmetry in the funnel plot (*p*-value = 0.0788). No evidence of publication bias was detected.

#### 3.1.4. AOFAS Scale

**Figure 14:**
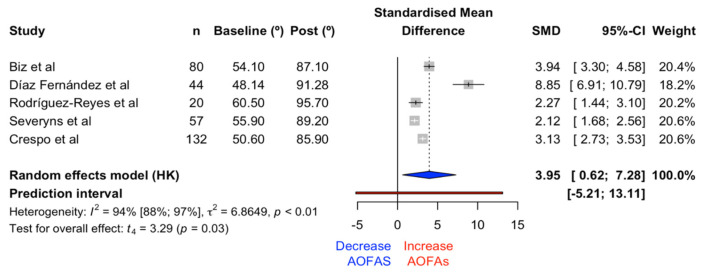
Forest plot for the AOFAS scale [19,22,24,25,27].

The meta-analysis on the effects of the AOFAS scale with the Reverdin–Isham osteotomy showed significant differences (t = 3.29, *p* = 0.03) in favor of an increase in the scale’s value, with an effect size of 3.95 (CI 0.62, 7.28). The forest plot of the AOFAS scale and its prediction interval is illustrated in Figure 14. The heterogeneity index was established at 93.5% (CI 87.8%, 96.6%).

**Figure 15:**
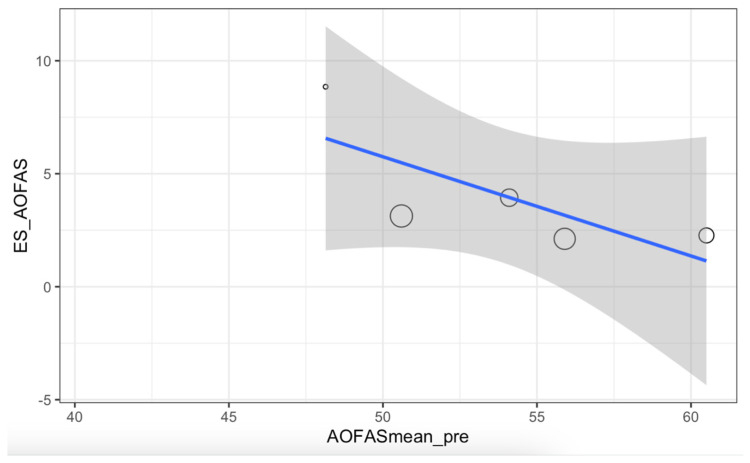
Meta-regression for the AOFAS scale.

The meta-regression for the AOFAS scale was not statistically significant (*p* = 0.054), with the relationship between the preoperative scale value and the achieved effect established at 41.98% (Figure 15).

**Figure 16:**
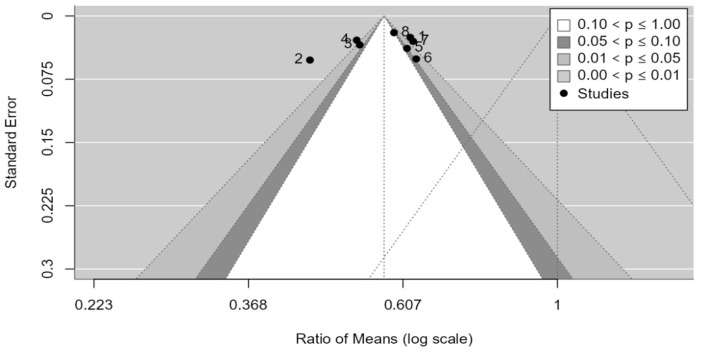
Funnel plot for the AOFAS scale.

As shown in the funnel plot (Figure 16), studies are distributed across all zones of significance, suggesting that publication bias is apparently not present, although the region with the highest *p*-values lacks studies, while studies are present in nonsignificant zones. Egger’s test confirms the absence of asymmetry in the funnel plot (*p*-value = 0.4183).

## 4. Discussion

The geographic origin of the included studies reflects a global diversity in the application and evaluation of this technique, with participation from countries such as Italy, Portugal, Spain, Mexico, Brazil, and France. This geographic diversity may have implications in terms of variability in clinical practices and patient characteristics, which should be considered when interpreting the results.

The quality assessment of the studies revealed a moderate level of quality, with a similarly moderate risk of bias. This suggests that, while the studies provide relevant information, it is important to interpret the results with caution and consider potential sources of bias that could influence the conclusions.

After reviewing the results of the analyzed literature [6,19,20,21,22,23,24,25,26,27], it is observed that all the included studies classify HV into mild and moderate categories. This expands the applicability of the Reverdin–Isham technique and its potential effectiveness across a broader range of cases. However, heterogeneity was identified in the classification systems used to measure HV across studies, and some did not describe the system used. This highlights the need to establish a standardized and protocolized classification system for future research to obtain homogeneous data and facilitate the comparison of results.

Some studies employed basal osteotomies for mild-to-moderate HV [22,27], an uncommon practice since these are generally reserved for more severe cases due to their greater corrective capacity, particularly in the IMA. Additionally, variability was observed in the measurement of radiological angles selected to evaluate the technique’s effectiveness. It is crucial that all studies using the Reverdin–Isham technique evaluate the PASA or DMAA both preoperatively and postoperatively, as their omission may limit the ability to adequately assess the technique’s efficacy.

Statistical analysis revealed considerable diversity in the results of the studies, with a notable lack of information in some cases, affecting the overall quality of the studies. The limited availability of statistical data and disparities in the presentation of information underscore the importance of standardizing the presentation of relevant statistical data in future studies to facilitate more accurate comparisons and more robust interpretation of the results.

The Reverdin–Isham osteotomy was employed in combination with various surgical techniques, varying according to the study and the surgeon’s preference. Despite the individual limitations of each study, a general mean angular improvement was observed across all evaluated angles, as well as an average increase in functionality and a decrease in pain among patients. This suggests a possible synergy between these complementary interventions and the main technique, highlighting the importance of a comprehensive and personalized approach to HV surgical management tailored to the specific characteristics of each patient.

The technique that combined the Reverdin–Isham osteotomy with adductor tenotomy, lateral capsulotomy, Akin osteotomy, and basal osteotomy demonstrated the best corrections in PASA, IMA, and HVA [22,27]. This combination is consistent, given that basal osteotomy is generally reserved for more severe HV cases, even though these studies [22,27] classified these cases as mild-to-moderate without specifying the classification system used. It is important to consider these results individually and not in conjunction with other techniques when analyzing the effectiveness of the Reverdin–Isham osteotomy in isolation.

In particular, the combination of Reverdin–Isham osteotomy with adductor tenotomy and Akin [26] showed the greatest correction in the PASA, with a mean improvement of 7.75 degrees. Regarding IMA and HVA, the combination with adductor tenotomy, lateral capsulotomy, and Akin [6,19,20,21,23,25] demonstrated the best angular correction, with 2.67 degrees and 14.41 degrees of mean improvement, respectively.

Significant improvements in functionality and pain reduction were observed in patients undergoing this technique, suggesting that the Reverdin–Isham osteotomy is effective in improving function and relieving pain in mild and moderate HV cases.

Regarding patients’ subjective perception, the results of foot function and pain level scales reflect a significant improvement in quality of life and postoperative well-being.

The high rate of personal satisfaction reported underscores the importance of considering not only the technical aspects of the surgery but also the overall impact on the patient experience and their perception of the treatment received. Patients expressed generalized satisfaction, and a high percentage would be willing to undergo the same surgical technique again.

This finding highlights the importance of minimally invasive surgery and supports its positive impact on HV surgical interventions and patient-related outcomes.

While the rates of major complications were generally low, with recurrences as the most common complication [19,20,22,25], the presence of this complication suggests the need for longer follow-up to identify and address it. Moreover, not all studies reported the absence of complications.

Most of the analyzed studies were retrospective [20,22,23,26], which poses inherent limitations. The lack of a prospective design, coupled with insufficient follow-up to detect complications, represents significant shortcomings.

Some of the reviewed studies included small sample sizes [20,21,23,24], which could affect the generalizability and validity of the results. Additionally, it is crucial to consider the experience and skill of the surgeon performing the intervention. One reviewed study [27] mentioned that the surgeon had limited experience, which could influence the results and constitute a bias due to variations in skill among professionals.

The shortening of the first metatarsal and the potential disruption of the Maestro line were neither mentioned nor analyzed in the reviewed studies, as they were not considered variables of interest. It is well known that transfer metatarsalgia is a common complication in hallux valgus correction procedures, particularly in certain surgical techniques. To minimize this risk, osteotomies of the lesser metatarsals are frequently performed as an adjunctive procedure. However, in the analyzed studies, transfer metatarsalgia is not reported as a complication, despite the fact that the performance of osteotomies in the lesser metatarsals as a preventive strategy is also not mentioned.

Regarding the meta-analysis results, a detailed analysis and careful interpretation are required.

First, regarding the PASA, the lack of statistical significance in reducing this angle and the high heterogeneity suggest that the efficacy of the Reverdin–Isham osteotomy may vary widely and is not consistently effective in all cases or conditions analyzed. Additionally, subgroup analysis and meta-regression did not provide clear evidence of factors that could significantly influence the results.

It is crucial to consider these findings in the clinical context, and it may be advisable to conduct additional studies with improved designs or specific focuses to better understand under what conditions this intervention might be most effective. This suggests that corrective effectiveness should be based on combining procedures depending on the severity.

The lack of statistical significance in the PASA results indicates that it cannot be confidently concluded that the Reverdin–Isham osteotomy consistently or significantly reduces this angle in the analyzed cases. This suggests that the effectiveness of this technique might be influenced by variations in clinical conditions, or the methodologies applied.

The high heterogeneity (98.4%) reflects considerable variability among the studies included in the meta-analysis, potentially due to differences in factors such as the studied populations, surgical protocols, or evaluation criteria. This heterogeneity limits the generalizability of the conclusions, as it suggests that the results are neither uniform nor replicable across different contexts. Consequently, these findings should be interpreted cautiously, with a focus on more specific analyses addressing the sources of heterogeneity.

For the IMA, a significant reduction was observed post-surgery. This may indicate that the Reverdin–Isham osteotomy has a consistent effect in correcting the IMA. The meta-regression showed a significant relationship between the preoperative angle and the achieved effect, meaning that the larger the preoperative angle, the greater the correction achieved through surgery. These results may be influenced by studies that combined basal osteotomies with Reverdin–Isham osteotomies in mild and moderate HV cases [22,27].

The HVA showed a significant reduction post-surgery, indicating an improvement in the alignment of the hallux relative to the first metatarsal. Although heterogeneity was also observed among the studies, the magnitude of the effect was considerable and statistically significant. However, meta-regression did not show a significant relationship between the preoperative angle and the achieved effect, suggesting that other factors might influence the correction of the HVA besides the initial angle value.

These radiographic results demonstrate positive points in angular correction with the Reverdin–Isham osteotomy. Systematic reviews comparing this technique also support these results [28,29,30], achieving significant correction.

Finally, regarding the AOFAS scale, a significant increase in the scale value was observed post-surgery. This indicates an improvement in overall foot function following the Reverdin–Isham osteotomy. Although heterogeneity among the studies and the wide confidence interval raise some uncertainty, the magnitude of the effect was considerable and statistically significant. However, the lack of statistical significance in meta-regression suggests that factors other than the preoperative scale value might influence foot function improvement post-surgery. These results align with other published studies, including meta-analyses [31], comparing minimally invasive surgery (MIS) with open surgery for HV, where the MIS group achieved higher AOFAS scores compared to open surgery.

Our analysis of the Reverdin–Isham osteotomy revealed a high probability of random effects. This is attributed to the poor scientific quality of the publications. Considerable diversity was observed in methodological descriptions, the number of operated feet, and the follow-up period. Additionally, demographic data were partially incomplete, and statistical information was imprecise. Only half of the studies included in the meta-analyses for PASA and AOFAS could be used, and only seven for IMA and HVA.

A significant limitation of this review stems from the various combinations of techniques associated with the analyzed osteotomy. The low scientific quality of studies on the Reverdin–Isham osteotomy found in the literature also represents a limitation. However, a positive aspect is the large total sample size of the review, with a total of 579 operated feet.

These results suggest that the Reverdin–Isham osteotomy may have variable effects on different aspects of HV. However, further research is needed to better understand the factors influencing these results and to identify which patients might benefit most from this surgical technique.

While these results provide an overview of the effects of the osteotomy on different aspects of HV, they also highlight the need to interpret them with caution due to the heterogeneity among studies. Future research with more rigorous study designs and larger sample sizes is needed to confirm and expand these findings.

Studies stratified by the combination of surgical techniques, conducted with more homogeneous samples in terms of age, sex, or concomitant pathologies, and considering the surgeon’s experience, are necessary to more deeply evaluate the effect of the Reverdin–Isham osteotomy in mild and moderate HV.

## 5. Conclusions

The findings of this study suggest that the Reverdin–Isham osteotomy may be an effective and safe surgical technique for the treatment of mild-to-moderate hallux valgus. The procedure appears to achieve improvements in the correction of the PASA or DMAAs, as well as in the intermetatarsal and hallux valgus angles, contributing to better anatomical alignment of the foot. However, given the limitations of the studies included, such as potential bias and high heterogeneity, these results should be interpreted with caution.

While recurrence was the most frequently reported complication, its clinical significance appears limited. Patients generally reported improved foot functionality, as measured by the AOFAS scale, and a notable reduction in pain according to the VAS, along with high satisfaction rates. Nevertheless, further robust studies are needed to confirm the effectiveness and safety of this minimally invasive technique in clinical practice.

## Figures and Tables

**Table 1:** Summary of the search strategy.

Database	Search Strategy	Total Results
PubMed	(Hallux valgus [Title/Abstract]) AND (Reverdin Isham [Title/Abstract] OR Oblique osteotomy [Title/Abstract])	25
SCOPUS	Article title, Abstract, Keywords (“Hallux valgus”) AND (“Reverdin Isham” OR “Oblique osteotomy”)	34
WOS	(TS = (“Hallux valgus”)) AND TS = (“Reverdin Isham” OR “Oblique osteotomy”)	39

## Data Availability

The raw data supporting the conclusions of this article will be made available by the authors on request.
